# Gliomatosis cerebri in children: A poor prognostic phenotype of diffuse gliomas with a distinct molecular profile

**DOI:** 10.1093/neuonc/noae080

**Published:** 2024-05-08

**Authors:** Gunther Nussbaumer, Martin Benesch, Yura Grabovska, Alan Mackay, David Castel, Jacques Grill, Marta M Alonso, Manila Antonelli, Simon Bailey, Joshua N Baugh, Veronica Biassoni, Mirjam Blattner-Johnson, Alberto Broniscer, Andrea Carai, Giovanna Stefania Colafati, Niclas Colditz, Selim Corbacioglu, Shauna Crampsie, Natacha Entz-Werle, Matthias Eyrich, Lea L Friker, Michael C Frühwald, Maria Luisa Garrè, Nicolas U Gerber, Felice Giangaspero, Maria J Gil-da-Costa, Norbert Graf, Darren Hargrave, Peter Hauser, Ulrich Herrlinger, Marion Hoffmann, Esther Hulleman, Elisa Izquierdo, Sandra Jacobs, Michael Karremann, Antonis Kattamis, Rejin Kebudi, Rolf-Dieter Kortmann, Robert Kwiecien, Maura Massimino, Angela Mastronuzzi, Evelina Miele, Giovanni Morana, Claudia M Noack, Virve Pentikainen, Thomas Perwein, Stefan M Pfister, Torsten Pietsch, Kleoniki Roka, Sabrina Rossi, Stefan Rutkowski, Elisabetta Schiavello, Clemens Seidel, Jaroslav Štěrba, Dominik Sturm, David Sumerauer, Anna Tacke, Sara Temelso, Chiara Valentini, Dannis van Vuurden, Pascale Varlet, Sophie E M Veldhuijzen van Zanten, Maria Vinci, André O von Bueren, Monika Warmuth-Metz, Pieter Wesseling, Maria Wiese, Johannes E A Wolff, Josef Zamecnik, Andrés Morales La Madrid, Brigitte Bison, Gerrit H Gielen, David T W Jones, Chris Jones, Christof M Kramm

**Affiliations:** Division of Pediatric Hematology and Oncology, Department of Pediatrics and Adolescent Medicine, Medical University of Graz, Graz, Austria; Division of Pediatric Hematology and Oncology, Department of Pediatrics and Adolescent Medicine, Medical University of Graz, Graz, Austria; Division of Molecular Pathology, Institute of Cancer Research, London, UK; Division of Molecular Pathology, Institute of Cancer Research, London, UK; U981, Molecular Predictors and New Targets in Oncology, Team Genomics and Oncogenesis of Pediatric Brain Tumors, INSERM, Gustave Roussy, Université Paris-Saclay, Villejuif, France; Department of Pediatric and Adolescent Oncology, Gustave Roussy, Université Paris-Saclay, Villejuif, France; U981, Molecular Predictors and New Targets in Oncology, Team Genomics and Oncogenesis of Pediatric Brain Tumors, INSERM, Gustave Roussy, Université Paris-Saclay, Villejuif, France; Department of Pediatric and Adolescent Oncology, Gustave Roussy, Université Paris-Saclay, Villejuif, France; Department of Pediatrics, Clínica Universidad de Navarra, Pamplona, Spain; Solid Tumor Program, Center for the Applied Medical Research, Pamplona, Navarra, Spain; Health Research Institute of Navarra (IdiSNA), Pamplona, Navarra, Spain; Department of Radiological, Oncological and Anatomo-Pathological Sciences, Sapienza University, Rome, Italy; Department of Paediatric Oncology, Sir James Spence Institute of Child Health, Royal Victoria Infirmary Queen Victoria Road, Newcastle upon Tyne, UK; Princess Máxima Center for Pediatric Oncology, Utrecht, The Netherlands; Pediatric Oncology Unit, Fondazione IRCCS Istituto Nazionale dei Tumori, Milano, Italy; Division of Pediatric Glioma Research, German Cancer Research Center (DKFZ) and German Cancer Consortium (DKTK), Heidelberg, Germany; Hopp Children’s Cancer Center (KiTZ), Heidelberg, Germany; Division of Hematology-Oncology, Children’s Hospital of Pittsburgh, Pittsburgh, PA, U.S.A; Neurosurgery Unit, Bambino Gesù Children’s Hospital (IRCCS), Rome, Italy; Oncological Neuroradiology and Advanced Diagnostics Unit, Bambino Gesù Children’s Hospital (IRCCS), Rome, Italy; Division of Pediatric Hematology and Oncology, University Medical Center Göttingen, Göttingen, Germany; Department of Pediatric Hematology, Oncology and Stem Cell Transplantation, University of Regensburg, Regensburg, Germany; Division of Molecular Pathology, Institute of Cancer Research, London, UK; UMR CNRS 7021—Laboratory of Bioimagery and Pathologies Team tumor signaling and therapeutic targets, University of Strasbourg, Illkirch, France; Pediatric Onco-Hematology Department—Pediatrics III, University Hospital of Strasbourg, Strasbourg, France; Pediatric Hematology and Oncology, Department of Pediatrics, University Hospital Würzburg, Würzburg, Germany; Institute of Neuropathology, DGNN Brain Tumor Reference Center, University of Bonn Medical Center, Bonn, Germany; Pediatric and Adolescent Medicine, Swabian Children’s Cancer Center, University Medical Center Augsburg, Augsburg, Germany; Neuro-Oncology Unit, IRCSS Istituto Giannina Gaslini, Genoa, Italy; Department of Oncology and Children’s Research Centre, University Children’s Hospital, Zurich, Switzerland; Department of Radiological, Oncological and Anatomo-Pathological Sciences, Sapienza University, Rome, Italy; Pediatric Oncology Department, University Hospital São João, Porto, Portugal; Department of Pediatric Oncology and Hematology, Saarland University, Homburg, Germany; Pediatric Oncology, Great Ormond Street Hospital for Children, London, UK; Second Department of Pediatrics, Semmelweis University, Budapest, Hungary; Division of Clinical Neuro-Oncology, Department of Neurology, University Hospital Bonn, Bonn, Germany; Division of Pediatric Hematology and Oncology, University Medical Center Göttingen, Göttingen, Germany; Princess Máxima Center for Pediatric Oncology, Utrecht, The Netherlands; Division of Molecular Pathology, Institute of Cancer Research, London, UK; Department of Pediatric Hematology and Oncology, University Hospitals Leuven, Belgium; Pediatric Oncology, Department of Oncology, KU Leuven, Belgium; Department of Pediatric and Adolescent Medicine, University Medical Center Mannheim, Medical Faculty Mannheim, Heidelberg University, Mannheim, Germany; Division of Pediatric Hematology-Oncology, First Department of Pediatrics, National and Kapodistrian University of Athens, “Aghia Sophia” Children’s Hospital, Athens, Greece; Division of Pediatric Hematology-Oncology, Institute of Oncology, Istanbul University, Istanbul, Turkey; Department of Radiation Oncology, University of Leipzig, Leipzig, Germany; Institute of Biostatistics and Clinical Research, University of Münster, Münster, Germany; Pediatric Oncology Unit, Fondazione IRCCS Istituto Nazionale dei Tumori, Milano, Italy; Department of Onco-Hematology, Gene and Cell Therapy, Bambino Gesù Children’s Hospital (IRCCS), Rome, Italy; Department of Onco-Hematology, Gene and Cell Therapy, Bambino Gesù Children’s Hospital (IRCCS), Rome, Italy; Department of Neurosciences, University of Turin, Turin, Italy; Center of Radiology, Department of Diagnostic and Interventional Radiology and Neuroradiology, Hospital of Fulda, Fulda, Germany; Division of Hematology-Oncology and Stem Cell Transplantation, Children’s Hospital, Helsinki University Hospital, Helsinki, Finland; Division of Pediatric Hematology and Oncology, Department of Pediatrics and Adolescent Medicine, Medical University of Graz, Graz, Austria; National Center for Tumor Diseases (NCT) Heidelberg, Heidelberg, Germany; Department of Pediatric Oncology, Hematology & Immunology, Heidelberg University Hospital, Heidelberg, Germany; Division of Pediatric Neurooncology, German Cancer Research Center (DKFZ) and German Cancer Consortium (DKTK), Heidelberg, Germany; Hopp Children’s Cancer Center (KiTZ), Heidelberg, Germany; Institute of Neuropathology, DGNN Brain Tumor Reference Center, University of Bonn Medical Center, Bonn, Germany; Division of Pediatric Hematology-Oncology, First Department of Pediatrics, National and Kapodistrian University of Athens, “Aghia Sophia” Children’s Hospital, Athens, Greece; Pathology Unit, Bambino Gesù Children’s Hospital (IRCCS), Rome, Italy; Department of Pediatric Hematology and Oncology, University Medical Center Hamburg-Eppendorf, Hamburg, Germany; Pediatric Oncology Unit, Fondazione IRCCS Istituto Nazionale dei Tumori, Milano, Italy; Department of Radiation Oncology, University of Leipzig, Leipzig, Germany; Department of Pediatric Oncology, University Hospital Brno and Faculty of Medicine, Masaryk University, Brno, Czech Republic; Department of Pediatric Oncology, Hematology & Immunology, Heidelberg University Hospital, Heidelberg, Germany; Division of Pediatric Glioma Research, German Cancer Research Center (DKFZ) and German Cancer Consortium (DKTK), Heidelberg, Germany; Hopp Children’s Cancer Center (KiTZ), Heidelberg, Germany; Department of Pediatric Hematology and Oncology, 2^nd^ Faculty of Medicine, Charles University in Prague and University Hospital Motol, Prague, Czech Republic; Division of Pediatric Hematology and Oncology, University Medical Center Göttingen, Göttingen, Germany; Division of Molecular Pathology, Institute of Cancer Research, London, UK; Department of Radiotherapy and Radiation Oncology, Faculty of Medicine and University Hospital Carl Gustav Carus, Technical University Dresden, Dresden, Germany; Princess Máxima Center for Pediatric Oncology, Utrecht, The Netherlands; GHU-Paris Psychiatry and Neuroscience, Sainte-Anne Hospital, Department of Neuropathology, Paris, France; Department of Radiology and Nuclear Medicine, Erasmus MC, Rotterdam, The Netherlands; Princess Máxima Center for Pediatric Oncology, Utrecht, The Netherlands; Department of Onco-Hematology, Gene and Cell Therapy, Bambino Gesù Children’s Hospital (IRCCS), Rome, Italy; Department of Pediatrics, Obstetrics and Gynecology, Division of Pediatric Hematology and Oncology, University Hospital Geneva, Geneva, Switzerland; Institute of Diagnostic and Interventional Neuroradiology, University of Würzburg, Würzburg, Germany; Department of Pathology, Amsterdam UMC, Amsterdam, The Netherlands; Princess Máxima Center for Pediatric Oncology, Utrecht, The Netherlands; Neurosurgery Unit, Bambino Gesù Children’s Hospital (IRCCS), Rome, Italy; AbbVie Inc, Oncology Development, North Chicago, IL, U.S.A; Department of Pathology and Molecular Medicine, 2nd Faculty of Medicine, Charles University in Prague and University Hospital Motol, Prague, Czech Republic; Pediatric Neuro-Oncology, Pediatric Cancer Center Barcelona, Hospital Sant Joan de Deu, Barcelona, Spain; Neuroradiological Reference Center for the Pediatric Brain Tumor (HIT) Studies of the German Society of Pediatric Oncology and Hematology, University Augsburg, Faculty of Medicine, Germany; Diagnostic and Interventional Neuroradiology, Faculty of Medicine, University of Augsburg, Augsburg, Germany; Institute of Neuropathology, DGNN Brain Tumor Reference Center, University of Bonn Medical Center, Bonn, Germany; Division of Pediatric Glioma Research, German Cancer Research Center (DKFZ) and German Cancer Consortium (DKTK), Heidelberg, Germany; Hopp Children’s Cancer Center (KiTZ), Heidelberg, Germany; Division of Molecular Pathology, Institute of Cancer Research, London, UK; Division of Pediatric Hematology and Oncology, University Medical Center Göttingen, Göttingen, Germany

**Keywords:** chromosome 6, gliomatosis cerebri, H3-wild-type and IDH-wild-type, pedHGG_RTK2, pediatric-type glioma, pediatric-type high-grade glioma

## Abstract

**Background:**

The term gliomatosis cerebri (GC), a radiology-defined highly infiltrating diffuse glioma, has been abandoned since molecular GC-associated features could not be established.

**Methods:**

We conducted a multinational retrospective study of 104 children and adolescents with GC providing comprehensive clinical and (epi-)genetic characterization.

**Results:**

Median overall survival (OS) was 15.5 months (interquartile range, 10.9–27.7) with a 2-year survival rate of 28%. Histopathological grading correlated significantly with median OS: CNS WHO grade II: 47.8 months (25.2–55.7); grade III: 15.9 months (11.4–26.3); grade IV: 10.4 months (8.8–14.4). By DNA methylation profiling (*n* = 49), most tumors were classified as pediatric-type diffuse high-grade glioma (pedHGG), H3-/IDH-wild-type (*n* = 31/49, 63.3%) with enriched subclasses pedHGG_RTK2 (*n* = 19), pedHGG_A/B (*n* = 6), and pedHGG_MYCN (*n* = 5), but only one pedHGG_RTK1 case. Within the pedHGG, H3-/IDH-wild-type subgroup, recurrent alterations in *EGFR* (*n* = 10) and *BCOR* (*n* = 9) were identified. Additionally, we observed structural aberrations in chromosome 6 in 16/49 tumors (32.7%) across tumor types. In the pedHGG, H3-/IDH-wild-type subgroup *TP53* alterations had a significant negative effect on OS.

**Conclusions:**

Contrary to previous studies, our representative pediatric GC study provides evidence that GC has a strong predilection to arise on the background of specific molecular features (especially pedHGG_RTK2, pedHGG_A/B, *EGFR* and *BCOR* mutations, chromosome 6 rearrangements).

## Teaser

For the podcast associated with this article, please visit ‘https://soc-neuro-onc.libsyn.com/gliomatosis-cerebri-in-children’

**Key Points**

- The presence of gliomatosis cerebri (GC) phenotype may be considered as an independent dismal prognostic factor in hemispheric pediatric-type diffuse gliomas.
- The methylome-based subtypes pediatric-type diffuse high-grade glioma (pedHGG)_RTK2A/B and (provisional) pedHGG_A/B were significantly associated with GC.
- *EGFR* and *BCOR* alterations and rearrangements of chromosome 6 were the most common genetic features in pediatric GC.

**Importance of the Study**

Gliomatosis cerebri (GC) is a rare, highly infiltrative phenotype of a diffuse glioma. Previous studies have failed to identify a molecular signature in GC compared with unselected gliomas in both children and adults. Our case series of more than 100 children and adolescents with GC represents a large GC cohort characterized by central radiological and pathological review. Several clinical (eg, treatment modalities, contrast enhancement), and histomolecular prognostic factors (eg, histopathological grading, presence of *TP53* mutations) were detected. For the first time, we were able to identify a molecular profile: the methylation subclasses pediatric-type diffuse high-grade glioma (pedHGG)_RTK2A/B and pedHGG_A/B were significantly associated with pediatric GC. Additionally, *EGFR* and *BCOR* alterations as well as rearrangements of chromosome 6 were the most common genetic features across tumor types. Taken together, these results may reopen a discussion on the nature of pediatric GC and provide insight into the disease biology of extensively infiltrating gliomas in children.

It is now widely acknowledged that pediatric diffuse gliomas differ fundamentally in key biological features compared to their adult counterparts.^1–5^ In recognition of these findings, the fifth edition of the World Health Organization (WHO) Classification of Tumors of the Central Nervous System (CNS) differentiates a priori between adult- and pediatric-type diffuse gliomas based on established molecular hallmarks.^6^ In this regard, methylome profiling has emerged as a remarkably robust and reproducible diagnostic tool for the classification of CNS tumors.^7,8^ However, this molecular-defined approach may neglect the clinical manifestations in various glioma (sub)types. For example, gliomatosis cerebri (GC) was originally defined as a diffuse glioma characterized by an extensively infiltrating growth pattern affecting at least 3 contiguous hemispheric lobes of the brain.^9^ With the revised fourth edition of the WHO classification, GC was no longer considered a distinct entity, but a phenotype characterized by a specific growth pattern of an underlying diffuse glioma without a distinct molecular signature.^10^ So far, similar (epi-)genetic characteristics were identified in tumors presenting a GC growth pattern as seen in various adult and pediatric types of non-GC gliomas of corresponding age groups.^11,12^ Since these data have been derived from adults or small pediatric case series, the biology of GC in children has not yet been conclusively defined. Thus, we conducted a multinational retrospective study of 104 children and adolescents with GC providing a comprehensive radiological, histopathological, clinical, and (epi-)genetic characterization.

## Methods

### Study Design

Following institutional review board (IRB number: 33–547 ex 20/21) approval by the Medical University of Graz, Austria, a Europe-wide multi-institutional, retrospective initiative was started to collect data of pediatric GC patients fulfilling the following inclusion criteria: (i) age <21 years at diagnosis, (ii) magnetic resonance imaging (MRI) at diagnosis showing a GC phenotype, (iii) neuropathological confirmation of diffuse glioma. Secondary GC following the progression of an initially localized glioma as well as primarily multifocal glioma were excluded. Fourteen European countries contributed to this study. For each included patient, either formalin-fixed paraffin-embedded and/or fresh-frozen tumor tissue was requested for histopathological and molecular analyses. The majority of methylation arrays and whole-exome sequencing (WES) were performed at the Institute of Cancer Research, London, in collaboration with the DKFZ, Heidelberg, for the purpose of this study. A subgroup (methylation array: *n* = 10; WES: *n* = 14) was performed at the Gustave Roussy Cancer Research Center, Paris.

### Neuroradiological Criteria

Central neuroradiological review was mandatory and performed in accordance with the 2007 WHO classification of CNS tumors,^9^ the last version in which GC was still described as a distinct entity. Only tumors showing a diffuse infiltrative process involving at least 3 contiguous cerebral lobes of the brain were included. The extent of infiltration was assessed on T2- or fluid-attenuated inversion recovery (FLAIR)-weighted MR-imaging. The lobus insularis was not counted as a separate lobe. All MRI scans were reviewed centrally by one of two experienced neuroradiologists (B.B. and M.W.-M.).

### Clinical Data

Apart from basic clinical parameters, clinical data included surgical procedures and nonsurgical oncological treatment. The type of resection was grouped into “biopsy” regardless of whether open or stereotactic, or “partial resection” if the extent exceeded pure diagnostic purposes acknowledging that effective debulking cannot be achieved in GC. Upfront treatment modalities were divided into radiotherapy, chemotherapy, or the combination of both. Because of the retrospective multicenter study design, chemotherapy regimens were highly heterogeneous. Therefore, data were harmonized by dividing them into 3 groups: “TMZ-Mono” if only temozolomide (TMZ) was applied, “TMZ-Multi” if TMZ was used in combination with other cytotoxic drugs or “Other” if chemotherapeutic regimes without TMZ were administered. Additionally, the administration of targeted therapies was recorded. The classification of cytotoxic therapies was performed regardless of dose, number of cycles, treatment duration, etc. Progression status was determined from local clinical and/or radiological reports.

### Neuropathological Assessment

Histopathological confirmation of a diffusely infiltrating glioma was mandatory. Data regarding histological tumor type and grade were extracted from local reports based on the WHO classification of tumors of the CNS at the time of diagnosis (WHO CNS classification 2007^9^ or 2016^10^). Therefore, Roman numerals have been retained here. WHO grade II tumors were summarized as “GC with low-grade features” (LGC), WHO grade III and IV tumors as “GC with high-grade features” (HGC). In a second step, all cases with available tumor material were centrally reviewed for the purpose of this study by 1 experienced neuropathologist or in the context of a neuropathological panel (constituted by G.H.G., P.V., T.Pi., F.G., S.Ro., M.A., J.Z., and P.W.) validating glial differentiation and grading. Reclassification according to WHO CNS 2021^6^ was performed retrospectively in synopsis with all molecular findings including DNA methylation profiles and the presence of pathognomonic mutations.

### Genome-Wide DNA Methylation Array Profiling

DNA methylation analysis was performed using either Illumina 450K or EPIC BeadArrays. Data were preprocessed using the *minfi* package [v1.46.0]^13^ and *mnp.v12b6* (DKFZ). The MNP12.5 random forest brain tumor classifier (molecularneuropathology.org) was used to assign a calibrated score to each case, associating it with 1 of 184 tumor types comprising the 2021 WHO Classification of CNS tumors and novel subclasses. DNA copy number was deduced from combined intensities using the *conumee* package [v1.34.0]^14^ as processed as combined log2 intensity data based upon an internal median processed using the R packages *minfi* and *conumee*. *t*-Distributed stochastic neighbor embedding (*t*-SNE) dimensionality reduction was carried out using the MNP12.5 classifier 10 000 training probes data. The *t*-SNE algorithm was applied using the *Rtsne* package [v0.16]^15^ on a distance matrix generated using 1-Pearson correlation, using default *Rtsne* package parameters except for “theta=0” and “max_iter=1000,” and primed with a numeric seed 12345 prior to execution for reproducibility. A clear allocation in *t*-SNE and/or a score of at least 0.6 was mandatory for subclass allocation.

### Whole-Exome Sequencing

For whole-exome sequencing, variants were called using GATK/mutect2 using current best practices. In summary, reads were aligned to GRCh37 using bwa [v0.7.17]^16^ and duplicates were removed with Picard Tools (http://broadinstitute.github.io/picard/) MarkDuplicates [v2.23.8]. After base quality score recalibration with GATK 4.1.9.0 variants were called with Mutect2 in joint calling mode including a panel of normal germline samples. Allelic depth was assigned by GATK VariantsToTable and variants were annotated for functional consequences with the ensembl variant effect predictor [v101.0].^17^ DNA copy number was calculated from normalized coverage using Picard Tools CollectHsMetrics and segmented with DNAcopy [v1.72.3].^18^ Integration of mutations and copy-number alterations into oncoprint representations was performed with custom scripts in R Studio 2023.03 and R version 4.2.2.^19^

### Statistical Analysis and Reference Cohorts

Data analysis was carried out with the software SPSS (v27)^20^ and R (v4.0.3)^19^ using a survival analysis package.^21^ Survival analyses were performed via Log-Rank test, Kaplan–Meier plots, and Cox-Regression (multivariate case). Comparisons between two groups regarding qualitative/quantitative variables were conducted using Fisher’s exact test/Mann–Whitney *U* test, respectively. In all analyses, alpha was set at 0.05 as the threshold for statistical significance. Since interpretations were explorative, adjustment of *P*-values for multiple testing was not performed. As a reference for comparison of molecular findings in GC, published data of adults and pediatric GC case series by Herrlinger et al.^11^ and Broniscer et al.^12^ were used. In both cohorts, methylation-derived data were updated and reclassified according to the MNP12.5 classifier. Published data on a population-based cohort of supratentorial diffuse gliomas in children (*n* = 80) by Sturm et al.^22^ served as a reference for comparison of the relative frequency of the different molecular glioma subtypes excluding nondiffuse methylation-defined neuroepithelial tumor subclasses, isolated midline location, and/or calibrated score <0.9. There was an overlap between these two collectives as two cases occurred in both the here-reported GC and this control cohort. As a reference for survival comparison in HGC, a cohort of unselected hemispheric HGGs in children (*n* = 108) extracted from the prospective German HIT trials (HIT-GBM-A/-B/-C/-D, interimGBM-D, HIT-HGG-2007/-2013) was compiled including tumors which were biopsied or partially resected only. Age limits were adjusted to those of the GC cohort. GC cases in the control group were excluded.

## Results

### Radiological, Clinical, and Histopathological Analyses Identify Prognostic Factors in Pediatric GC

**Figure 1:**
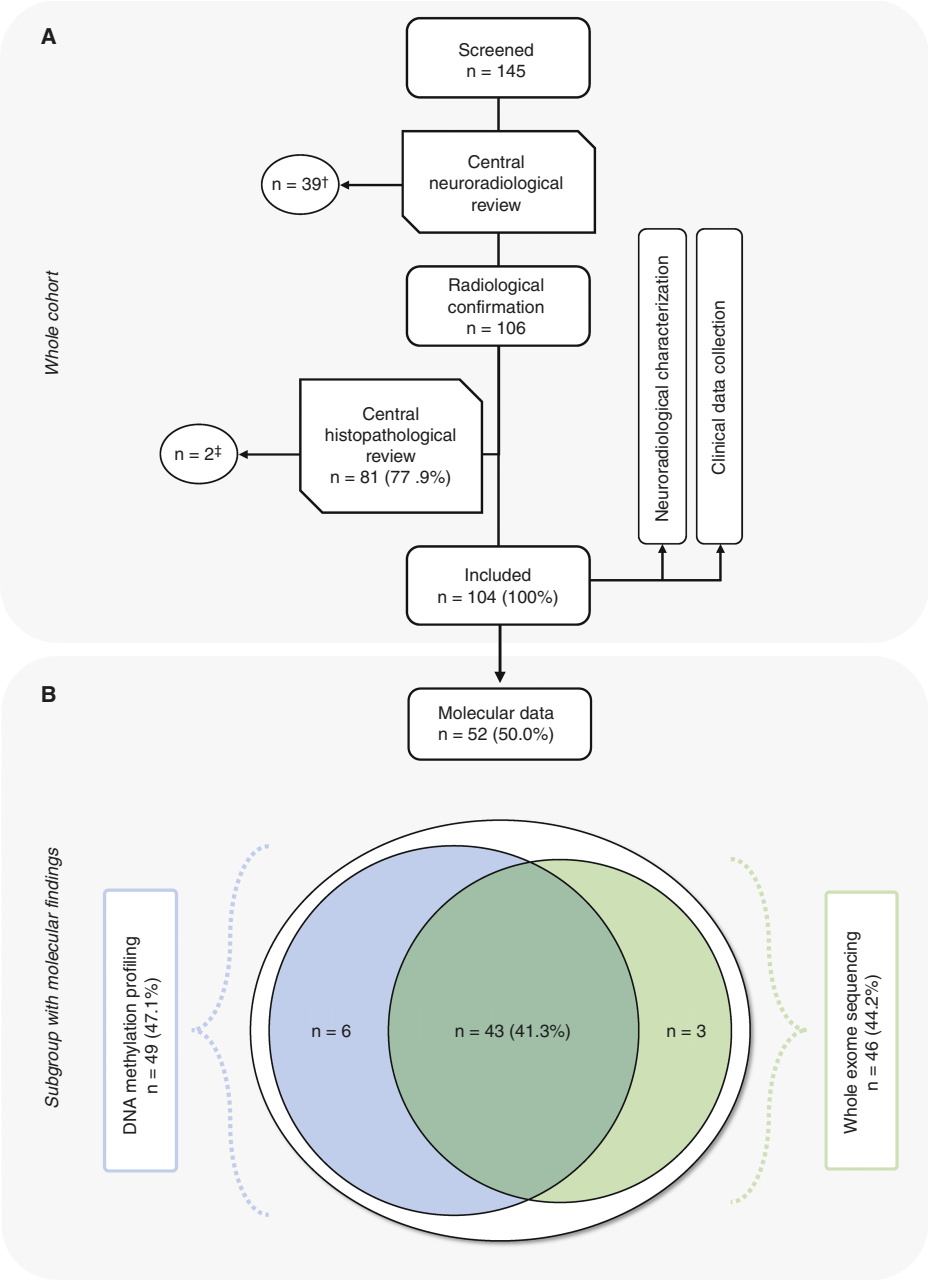
(A) Flowchart of the study cohort. (A) A total of 145 children and adolescents from 14 European countries were screened for suspected GC. ^†^39 cases did not fulfill the neuroradiological criteria and were excluded. ^‡^Two tumors were excluded as a glial process could not be confirmed unambiguously through central neuropathological review. (B) Composition of the subgroup with available molecular data comprising DNA methylation profiling and whole exome sequencing.

**Figure 2:**
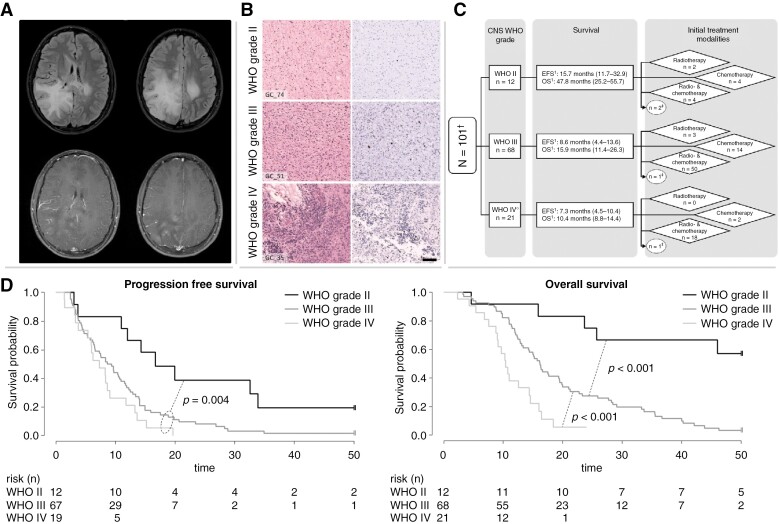
Collage of clinical data of the whole cohort. (A) Representative MRI of a pediatric patient with GC. Upper row: Fluid-attenuated inversion recovery (FLAIR): high signal as a sign of diffuse tumor infiltration primarily in the right occipital, parietal, and temporal lobe as well as involvement of the contralateral hemisphere with little mass effect. Lower row: contrast-enhanced T1-weighted images: the occipital part of the tumor shows typical mild multifocal enhancement. (B) Histopathological features of GC. Microscopical examination of three representative GC tumors according to histopathological grading: the left side shows HE staining and the right Ki-67 immunohistochemistry of the respective case: GC_74, WHO grade II; GC_51, WHO grade III; GC_35, WHO grade IV. With higher WHO grade, increasing cellularity and proliferative activity can be detected. Scale bar equate to 100 µm in each case. (C) Composition of the treatment groups according to the WHO grade. ^1^PFS and OS given as median and the interquartile range in parentheses. ^†^Three cases were excluded for this analysis due to absent WHO grading. ^‡^These patients did not receive an upfront cytotoxic treatment. (D) Kaplan–Meier plot including *p*-value of the (left) PFS and (right) OS in months according to WHO grading.

From screening 145 patients, a total of 104 children and adolescents with GC were included (Figure 1A). In most patients (*n* = 95, 91.5%), 3–5 cerebral lobes were affected (range 3–8). In 56 cases (53.8%), both cerebral hemispheres were involved. An additional uni- or bilateral involvement of the thalamus was observed in 48/30 (46.1%/28.9%) patients, respectively. Contrast enhancement was absent in 50 patients (48.1%) and in 49 children (47.1%) a predominantly focal enhancement was present. A representative MRI is displayed in Figure 2A.

The median age at diagnosis was 11.8 years (range 1.3–18.8) with a male predominance of approximately 2:1. Seizures were the most frequent symptom (*n* = 48, 46.2%). Most tumors (*n* = 68, 65.4%) were described as histopathological WHO grade III, followed by grade IV (*n* = 21, 20.2%) and grade II (*n* = 12, 11.5%) according to the WHO classifications of CNS tumors of 2007 or 2016. Respective photographs of the histopathologic grades in GC are shown in Figure 2B. Overall, central neuropathological review was performed in 81 tumors (77.9%).

Seventy-nine children (75.9%) underwent initial radiotherapy. Ninety-four children (90.4%) received upfront chemotherapy (mono/combined) and, hereof, monotherapy with temozolomide was most frequently used (*n* = 52, 50.0%). A primary combined modality treatment of chemotherapy and radiotherapy (parallel and/or consecutive) was performed in 73 children (70.2%). Twelve patients (11.5%) received upfront targeted therapies such as anti-EGFR (*n* = 7) or anti-VEGF (*n* = 5), mostly in combination with other agents, for example, Vinorelbine or TMZ. Eight children (7.7%) underwent re-irradiation. Comprehensive radiological and clinical characteristics are summarized in Supplementary Tables 1 and 2A.

At the last follow-up, 7 patients (6.8%) were alive with a median follow-up of 83.1 months (range 13.1–138.8), and 4 of them (3.8%) survived >5 years after diagnosis. Ninety-three children (89.4%) died due to disease progression. The median progression-free (PFS) and overall survival (OS) were 8.6 months (interquartile range [Q1–Q3]: 4.3–14.0) and 15.5 months (Q1–Q3: 10.9–27.7) with 1-year and 2-year OS rates of 68.0% and 28.1%, respectively (Supplementary Table 2B).

In univariate analysis, none of the following parameters were significantly associated with PFS or OS: number of affected cerebral lobes (<5 vs. ≥5), bihemispheric or infratentorial involvement, bilateral involvement of basal ganglia, contrast enhancement, sex, age at diagnosis (±4 and ±10 years of age). However, patients with bithalamic involvement showed significantly shorter PFS (median PFS: 6.5 months [Q1–Q3: 3.0–14.0] vs. 10.4 months [Q1–Q3: 5.7–14.9]; *P* = .025) and a tendency toward decreased overall survival (*P* = .059). Furthermore, WHO grade was significantly associated with PFS (*P* = .004) and OS (*P* < .001) (Figure 2D). Median OS of patients with histological low- (WHO grade II [≙ “LGC”]) and high-grade features (WHO grade III/IV [≙ ‘HGC’]) was 52.4 (Q1–Q3: 23.7–59.0) and 14.6 months (Q1–Q3: 10.4–21.2), respectively. In HGC, contrast enhancement was associated with inferior PFS (median PFS: 6.2 months [Q1–Q3: 3.6–11.7] vs. 10.7 months [Q1–Q3: 6.3–14.9]; *P* = .044). Patients with high-grade tumors were more commonly treated by combined modality approaches during first-line therapy compared to patients with WHO grade II tumors (*P* = .017) (Figure 2C). Treatment modalities had a significant impact on PFS in the HGC subgroup: combination of radio- and chemotherapy was associated with longer PFS compared to chemotherapy alone (median PFS: 9.6 months [Q1–Q3: 5.7–14.0] vs. 4.3 months [Q1–Q3: 3.0–7.4]; *P* < .001), whereas the administration of targeted therapy or total irradiation dose (<50 vs. >50 Gy) did not affect PFS in the whole cohort. By contrast, in terms of treatment modalities, no difference in PFS was observed in the LGC subgroup. However, neither in the HGC nor in the LGC subgroup, OS was significantly influenced by the treatment strategy including re-irradiation or targeted therapies (VEGF or EGFR inhibition) in the respective histopathological subgroup. Multivariate analysis of the whole cohort confirmed the impact of WHO grade on PFS and OS, as well as that of treatment groups and contrast enhancement on PFS (Supplementary Table 3).

To determine whether GC phenotype may represent an independent prognostic parameter, the HGC cohort was compared with a reference collective composed of hemispheric HGGs from the German pedHGG study cohorts. Since GC by nature cannot be completely resected, and the extent of resection in pedHGG is a recognized prognostic parameter,^23,24^ for better comparability, we only included hemispheric tumors that were partially resected at best in the control group. Compared to this reference cohort (*n* = 108), HGC showed inferior overall survival (median OS: 14.6 months [Q1–Q3: 10.4–21.2] vs. 16.5 months [Q1–Q3: 11.9–34.8]; *P* = .007). GC phenotype, extent of resection, and histopathologic grading remained statistically significant in multivariate testing (Supplementary Figure 1 A and 1B).

### Accumulation of Certain DNA Methylation-Based Subclasses and Chromosome 6 Alterations

Within our cohort of pediatric GC, tumor material was available from 52 patients (50.0%), of which 49 were subjected to DNA methylation profiling and 46 to whole-exome sequencing (WES); in 43 cases (41.3%), both DNA methylation and WES were conducted (Figure 1B).

**Figure 3:**
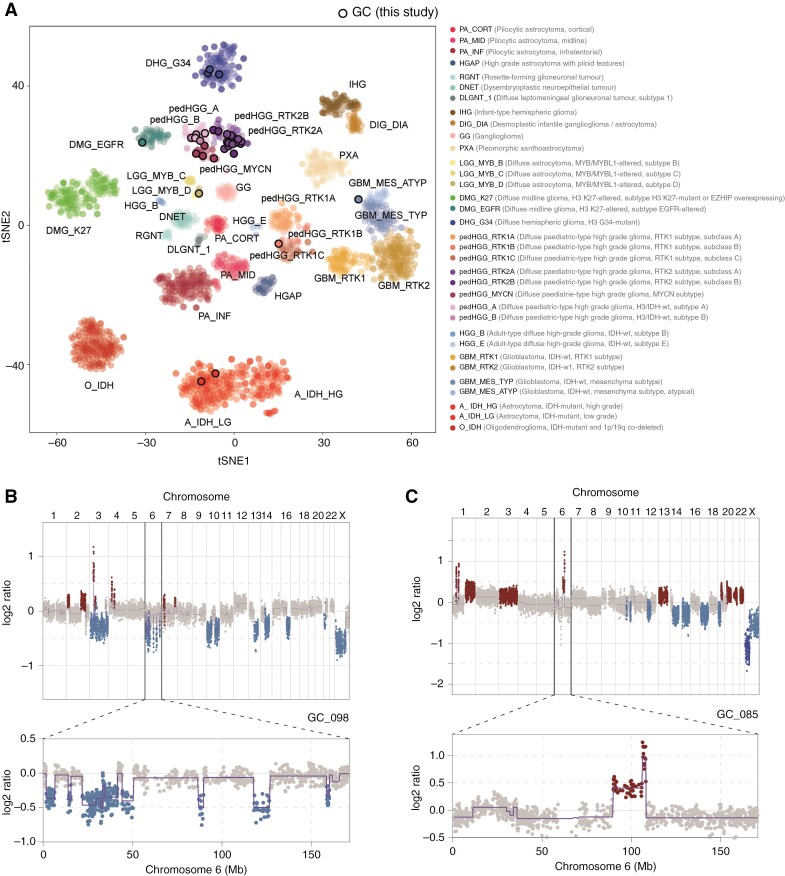
DNA methylation profiling in pediatric GC. (A) *t*-Statistic-based stochastic neighbor embedding (*t*-SNE) projection of a combined methylation dataset according to the MNP12.5 classifier comprising the pediatric GC cases from this study (circled, *n* = 40/49) plus a reference set of glioma subtypes (*n* = 2305). The first 2 projections are plotted on the *x* and *y* axes, with samples represented by dots colored by the respective subclass as labeled on the figure. (B, C) DNA copy-number plots for the cases GC_098 and GC_085 derived from methylation array data, with log2 ratios plotted (*y* axis) against genomic location by chromosome (*x* axis), and colored red for gain, and blue for loss. Above—whole genome; below—chromosome 6 illustrating representing examples of the structural alteration observed in our cohort.

GC tumors analyzed by methylation arrays underwent subclassification by the MNP12.5 classifier and were projected by *t*-distributed stochastic neighbor embedding (*t*-SNE) alongside a reference background of *n* = 2305 gliomas^7,25–28^ of all ages, grades, classes, and subclasses (Figure 3A). A total of 9 GC cases (9/49, 18.4%) were excluded due to poor classification scores (<0.6), and/or inconsistent clustering by *t*-SNE, hereafter described as “NEC” (Not Elsewhere Classified). The vast majority of successfully allocated tumors (30/40, 75.0%) were assigned to the closely clustering subclasses of pedHGG_RTK2A/B (*n* = 16 + 3, 47.5%), pedHGG_MYCN (*n* = 5, 12.5%), or pedHGG_A/B (*n* = 2 + 4, 15.0%). The latter are considered novel, not yet published (provisional) molecular subclasses according to MNP12.5.^7^ There were 3 additional tumors (7.5%) of the pediatric-type DHG_G34, and individual cases (*n* = 1, 2.5% each) representing the midline DMG_EGFR subclass, or MYB(L1)-altered diffuse glioma, subtype D. Notably, there was only 1 tumor of the pedHGG_RTK1A/B/C subclass of a child, who had received previous CNS irradiation for leukemia. The remaining 4 tumors (10.0%) presented adult-type entities clustering as follows: A_IDH_LG (*n* = 2), GBM_RTK2 (*n* = 1), and GBM_MES_ATYP (*n* = 1).

**Figure 4:**
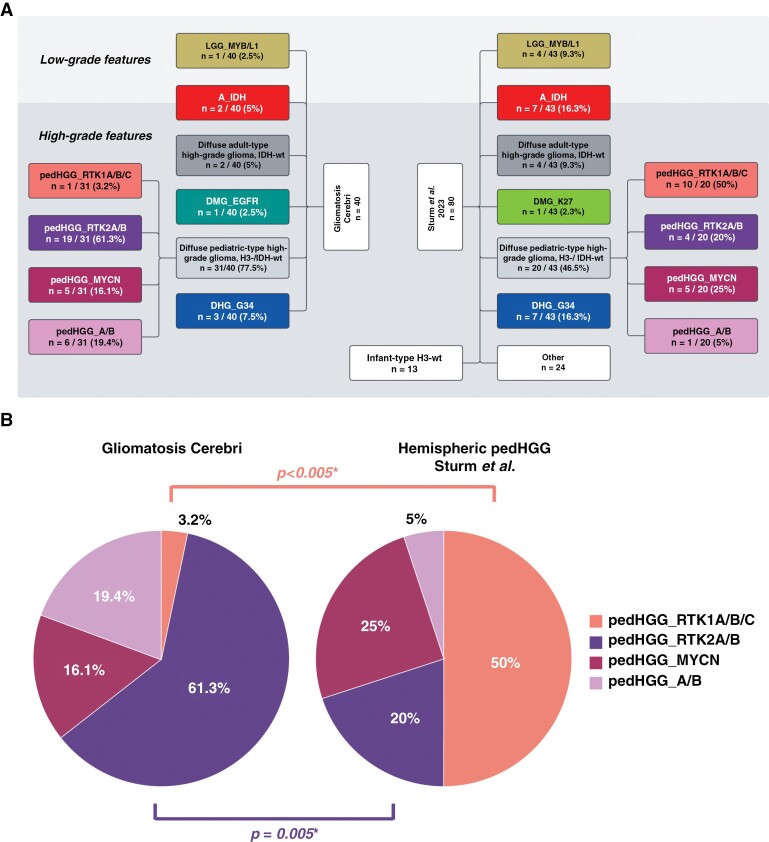
Distribution of methylation-based subclasses. (A) Comparison of DNA methylation data according to MNP12.5 of the GC cohort (*n* = 40) and the published population-based collective by Sturm et al.^22^ (*n* = 80). The subclasses were arranged based on the WHO CNS 2021 classification. In the cohort by Sturm et al., only supratentorial cases with hemispheric location were included. All non-diffuse glioma subclasses, isolated midline location, and with a calibrated score <0.9 were excluded. Infant-type H3-wild-type tumors were not considered in the analysis to approximate the age distribution between the 2 cohorts (median 12 years [1–21] vs. 11.8 years [1.3–18.8]). In the category “others” 22 tumors of “pleomorphic xanthoastrocytoma (-like)” and 2 tumors of the subclass “neuroepithelial tumor, PLAGL1-fused” were present. There was an overlap between these 2 collectives as 2 cases occurred in both the here-reported GC and the control cohort. Therefore, in the subclasses pedHGG_A/B and pedHGG_RTK2A/B, 1 patient is listed once in each of the 2 cohorts. Of note, the only pedHGG_A/B-case in the reference collective was a child from the here-presented GC cohort. (B) Relative frequencies of different subclasses in the diffuse pediatric-type high-grade glioma, H3-wild-type, and IDH-wild-type subgroup of the 2 above-mentioned cohorts. *There was a significant difference in the frequencies of pedHGG_RTK2A/B- and pedHGG_RTK1A/B/C subclasses between the 2 cohorts.

Compared with the population-based cohort of methylome-defined hemispheric diffuse gliomas by Sturm et al.^22^ (*n* = 80), there were distinct differences in the subclass distributions between the 2 collectives: apart from the absence of hemispheric infant-type, H3-wild-type (wt) tumors in the GC collective, the reference cohort showed a significantly increased frequency of pedHGG_RTK1 cases (10/20 vs. 1/31, *P* < .001) while tumors of the pedHGG_RTK2A/B subclass were almost absent (1/20 vs. 19/31, *P* = .005). Due to the small number of samples, no meaningful comparison regarding the pedHGG_MYCN and pedHGG_A/B subclass could be drawn (Figures 4A and 4B).

In DNA copy-number profiles generated from the methylation array data, common alterations seen in pedHGG such as gain of chromosome 1q (11/49, 22.4%) were present in several cases of different methylome-defined subclasses. A significant proportion of gliomas harbored structural alterations of chromosome 6 (18/49, 36.7%). These included cases with highly complex rearrangements, partial or whole arm losses, or gains/amplifications (Figure 3B and 3C). These aberrations were found in 5/6 tumors classified as pedHGG_A/B as well as in several other subclasses, but were mutually exclusive with the pedHGG_RTK2A subclass (0/16), which harbored relatively few copy-number alterations in general (Supplementary Figure 2).

### Comparison Between Different GC Cohorts Confirms Prevalence of Certain Subclasses

To determine the applicability of these results in other GC datasets, methylation array data from published case series of 18 pediatric^12^ and 25 adult GC cases^11^ were reclassified according to MNP12.5: in the pediatric set (median age: 11 years, range: 1–19), a similar pattern of molecular profiles was observed as in the present GC cohort: besides IDH-mutant (*n* = 3), DHG_G34 (*n* = 2), and DMG_EGFR (*n* = 1) subclasses, the remaining classifiable cases clustered as either pedHGG_RTK2A/B (*n* = 6), pedHGG_A (*n* = 1), or pedHGG_MYCN (*n* = 1). Likewise, no cases of pedHGG_RTK1A/B/C group were detected in the pediatric GC-control group (Supplementary Figure 3A). By contrast, in the adult series (median age: 50 years, range 24–77), an enrichment in age-typical subclasses such as A_- or O_IDH (*n* = 11), GBM_RTK1 (*n* = 4), and GBM_MES_TYP (n = 3) was present. Interestingly, within the adult cohort, there was 1 case of pedHGG_B in a 39-year-old GC patient (Supplementary Figure 3B). Notably, cases in both series (pediatric 4/18, 22.2%; adult 3/25, 12.0%) were also found to harbor structural chromosome 6 alterations including 1 pedHGG_B tumor, which was considered as chromothripsis, whereas the general pattern of DNA copy-number changes reflected the wider diffuse glioma landscapes in the respective age groups (ie, 1q+ in children, 7+/10− in adults) (Supplementary Figure 4A and 4B).

### Enrichment of *EGFR* and *BCOR* Alteration in Pediatric GC

**Figure 5:**
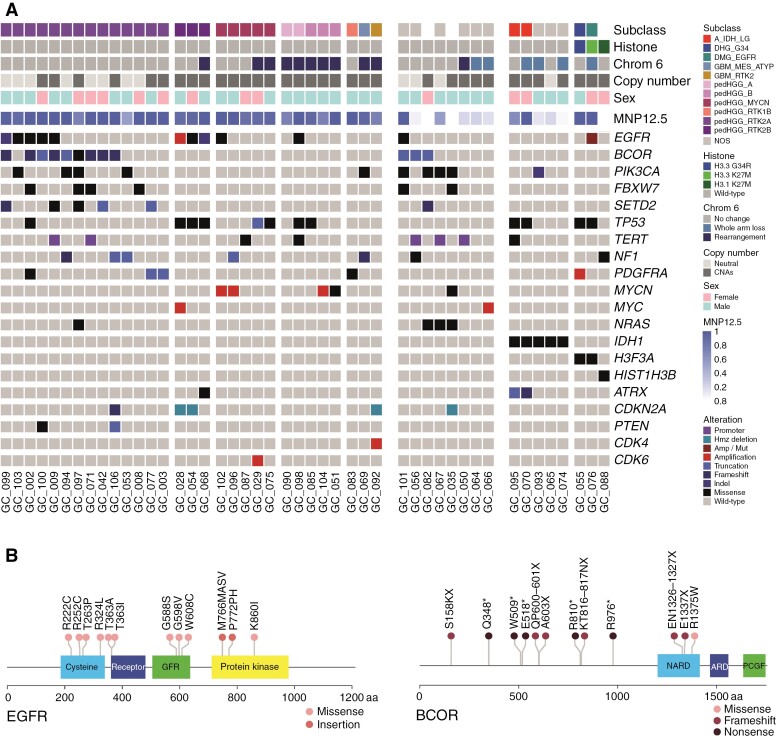
Whole-exome sequencing of pediatric GC. (A) Oncoprint representation of an integrated annotation of single-nucleotide variants and DNA copy-number changes for pediatric GC (*n* = 46). Samples are arranged in columns with genes labeled along rows. Clinicopathological and molecular annotations are provided as bars according to the included key. (B) Lollipop plot of specific variants identified in pediatric GC cases in *EGFR* (left) and *BCOR* (right), scaled by number and colored according to the key provided. Abbreviations: cysteine-rich domain (cysteine); receptor L-domain (receptor); growth factor receptor domain 4 (GFR); protein kinase domain (protein kinase); non-ankyrin-repeat domain (NARD); ankyrin repeat domain (ARD); PCGF1-binding domain (PCGF).

Exome sequencing identified subtype-specific alterations enriched in our GC series (Figure 5A). In addition to pathognomonic mutations in specific diffuse glioma subtypes (*n* = 5 of *IDH_*R132H, *n* = 1 each of *H3F3A*_G34R, *H3F3A*_K27M, *HIST1H3B_*K27M), a low frequency of common alterations in hemispheric pedHGG, such as *CDKN2A/B* deletion (4/46, 8.7%), *ATRX* mutation/deletion (3/46, 6.5%), or *PDGFRA* activating mutation/amplification (3/46, 6.5%) was detected. Alterations (mainly missense mutations) in *EGFR* were found in 12/46 (26.1%) tumors, particularly in the pedHGG_RTK2A/B subgroup (8/17, 47.1%), and were largely found in the extracellular domains, though with no recurrent hotspot mutations (Figure 5B). The equally most common mutated genes were *TP53* and *BCOR*, with predicted inactivating mutations found in 12 cases each (26.1%). Three-quarters of *BCOR* alterations (*n* = 9) were present in pedHGG_RTK2A, the other 3 in the NEC subgroup. Additional common mutations in the pedHGG_RTK2A subgroup included *PIK3CA* (*n* = 4; plus 4 in NEC), *FBXW7* (*n* = 4 + 2) and *SETD2* (*n* = 5 + 1) (Figure 5A).

### Synthesis of Molecular Data According to WHO CNS 2021

**Figure 6:**
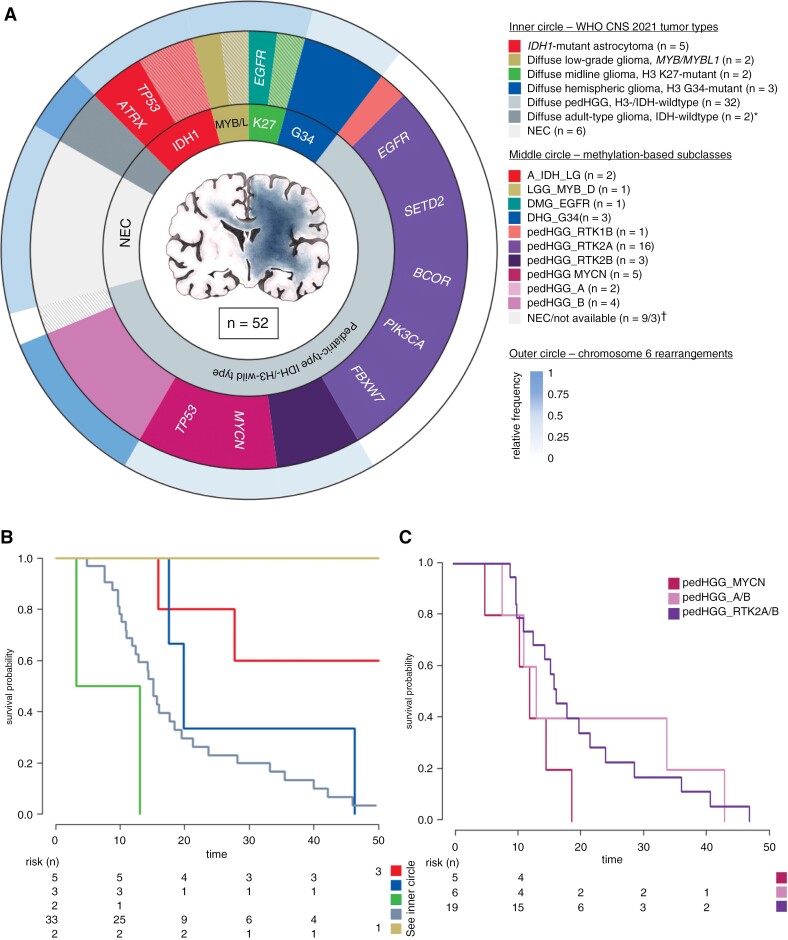
Summary of the molecular findings in a multilayer circle diagram. (A) In total, molecular data was available of 52 tumors. *The inner circle* represents different tumor types according to WHO CNS 2021 in synopsis with methylation array data and exome sequencing. *The middle circle* illustrates the DNA methylation-based subtypes according to the MNP12.5 classifier, which clustered to the corresponding WHO CNS types. In addition, the most frequent genetic alterations of the respective subtypes are given. In both circles the (sub-)types are colored as labeled in the given key. Hatched areas represent cases with inconclusive methylation profiling, but subtype allocation was possible through detection of disease-defining genetic alterations in exome sequencing. *adult-type diffuse gliomas, IDH-wild-type (*n* = 2) including 1 case each of the subtypes GBM_MES_ATYP and GBM_RTK2. ^†^Nine cases were NEC in methylation analyses, and additionally, in 3 cases, no methylation data were available. *The outer circle* represents the relative frequency of chromosome 6 rearrangements sorted by the respective methylome-based subclass. (B, C) Kaplan–Meier plots of the overall survival in months according to (B) the WHO CNS 2021 and to (C) the methylation-defined subclasses of the pedHGG_H3-/IDH-wt subgroup including pedHGG_A/B. Tumors of the adult-type, IDH-wild-type are not shown in (B).

Subsequently, all tumors with molecular data available (*n* = 52) were reclassified using the integrated diagnostic criteria according to the WHO classification of CNS tumors of 2021,^6^ based on methylation arrays and the presence of pathognomonic mutations. The majority of tumors (including patients with the provisional pedHGG_A/B subclass) were classified as diffuse pedHGG, H3-wt, and IDH-wt (pedHGG_H3-/IDH-wt) (*n* = 32/52, 61.5%), followed by astrocytoma, *IDH1*-mutant (*n* = 5, 9.6%), and diffuse hemispheric glioma, H3 G34-mutant (*n* = 3, 5.8%). Two tumors each (3.8%) belonged to the diffuse midline glioma, H3 K27-altered, the diffuse LGG, *MYB/MYBL1*-altered, and the adult-type glioblastoma, IDH-wt, tumor (sub-)types, respectively. In none of the *IDH1*-altered tumor samples, oligodendroglial features or 1p/19q-codeletion were present. Six tumors (11.5%) were still considered as NEC. A summary of the molecular findings is shown in Figure 6A. Compared with the pedHGG_H3-/IDH-wt subtype (median OS: 15.2 months [Q1–Q3: 10.9–23.7]), *IDH1*-mutant gliomas showed prolonged survival (median OS: 54.6 months [Q1–Q3: 27.7–131.2]), whereas the number of cases is too small to draw a statistical conclusion (Figure 6B). Of the 9 LGC tumors available for molecular analysis, 4 cases were *IDH1*-mutant tumors, 2 cases turned out to be pedHGG_RTK2A, and 1 was *MYB/MYBL*-altered (2 tumors were still classified as NEC).

### Characteristics of the pedHGG_H3-/IDH-wt Subgroup With GC Phenotype

In the pedHGG_H3-/IDH-wt subgroup, 29 of the 32 patients (90.6%) succumbed to their disease. Within this cohort, several prognostic factors were observed: with a median age of 12.1 years [Q1–Q3: 8.1–14.2], children under 10 years of age (*n* = 10) showed decreased OS compared to older patients (median OS 10.3 months [Q1–Q3: 9.7–14.4] vs. 17.7 months [Q1–Q3: 12.4–33.2]; *P* =* *.004). With regard to the 3 different subtypes, patients of the pedHGG_MYCN subtype were younger than patients with pedHGG_RTK2A/B- and pedHGG_A/B gliomas (median age 5.3 years [range: 2.9–7.8] vs. 12.8 years [range 6.3–18.0]). Cases of pedHGG_RTK2A showed a tendency for a more extensive growth pattern at diagnosis with a median of 5 affected cerebral lobes (Q1–Q3: 4–5) versus 3 lobes ([Q1–Q3: 3–5] in both other subclasses (*P* = .073) (Supplementary Figure 5). In univariate analysis, neither the different methylation-based subtypes (pedHGG_RTK2A/B vs. pedHGG_MYCN vs. pedHGG_A/B) (Figure 6C), nor the presence of structural alterations of chromosome 6 were associated with survival. Of all WES-derived molecular alterations tested (including *EGFR*, *BCOR*, *PIK3CA*, *FBXW7*, *SETD2*, *MYCN*), only the presence of *TP53* alterations had a significant negative effect on OS (median OS: 9.7 [Q1–Q3: 8.8–12.4] vs. 18.4 months [Q1–Q3: 14.2–28.2]; *P* < .001). Due to the small number of cases, the statistical analysis of the impact of administrating VEGF or EGFR inhibitors in the respective methylation-based subgroup or in *EGFR*-altered tumors could not be investigated in a meaningful way. In multivariate analysis of the pedHGG_H3-/IDH-wt subgroup comprising age, methylation subclass, and *TP53*-status, only the latter remained to affect OS significantly (*P* = .008) (Supplementary Table 4).

## Discussion

This large multi-institutional study on childhood GC aimed at improving the knowledge of this exceptional phenotype of diffuse gliomas. In comparison to hemispheric pedHGG, we showed that GC growth pattern per se is associated with an inferior prognosis. Grading according to the WHO classification of CNS tumors 2007/2016 demonstrated a significant impact on outcome: conventional histological grading was found to be an overall and HGC-related prognostic marker, whereas the absence of histopathological high-grade features was associated with a survival benefit. We also demonstrated that GC with low-grade features does not behave clinically like pediatric low-grade glioma (pedLGG) since the majority of children with LGC succumbed eventually to tumor progression, whereas pedLGGs are associated with low mortality in general.^29,30^ In molecular analyses, besides MYB/L-altered tumors, no pathognomic pedLGG alteration could be identified. On the contrary, 4 of the 9 LGC tumors available for workup were IDH-altered, which underlines the different molecular origin between pedLGG and LGC. The presence of MYB/L- and IDH-altered tumors, which are associated with more favorable survival in diffuse glioma in children/adolescents,^31–34^ might contribute to the prolonged survival in the LGC subgroup. In summary, with respect to mortality rates and our scarce molecular data in this subgroup, we support the concept that GC with histological low-grade characteristics—even in the absence of molecular high-grade features—should be considered high-grade entities and approached as such. The adequate treatment strategy for MYB/L or IDH-altered tumors with GC phenotype remains to be determined.

We demonstrated that certain subtypes of diffuse gliomas as defined by the WHO CNS 2021 classification are significantly associated with a GC phenotype. Diffuse pediatric-type HGG, H3-wt and IDH-wt, was the most common WHO type, consisting mainly of DNA methylation subclasses pedHGG_RTK2A/B, pedHGG_A/B, and pedHGG_MYCN. Except for single clinical parameters (eg, younger age in pedHGG_MYCN), these subclasses were associated with similar clinical phenotypes and cluster closely on *t*-SNE. Interestingly, gliomas of the pedHGG_RTK1A/B/C subclass, which may account for approximately one-third in unselected pedHGG_H3-/IDH-wt cases^35^ and accounted for half in our control group,^22^ were virtually absent in our series as well as in another independent pediatric GC cohort published by Broniscer et al.^12^

Besides, our study indicates that the accumulation of some genetic aberrations may promote a GC phenotype as well. For example, gene aberrations in *EGFR* and *BCOR* were found at unexpectedly high frequencies in our pediatric GC series. Within published hemispheric cohorts of pedHGG, the frequency of *EGFR* mutations was 11/326 (3.4%)^25^ and 2/86 (2.3%).^26^ Unselected pedHGG_H3-/IDH-wt tumors may show *EGFR* amplifications,^35^ but *EGFR* mutations as described in our series are uncommon outside of the newly recognized DMG-EGFR methylation-based subclass.^36,37^ These diffusely infiltrating (often bithalamic) pediatric diffuse midline gliomas show phenotypic similarities with GC. However, unlike DMG-EGFR characterized by hotspot *EGFR* mutations, in our *EGFR*-mutated GC tumors, the mutations did not cluster in specific regions of the gene. *BCOR* and *FBXW7* alterations have previously been described mainly in H3-altered HGGs,^25^ and *SETD2* alterations in hemispheric H3-wt pedHGG.^38^ Whether alterations in *BCOR*, *PIK3CA*, *FBXW7*, or *SETD2*, which occurred mainly in our pedHGG_RTK2A subgroup, are significantly associated with this specific methylome-based subclass or with GC per se, has to be clarified in future studies with a higher number of tumors. Korshunov et al.^35^ published a small, unselected cohort of pedHGG_RTK2 cases (*n* = 18), which were primarily supratentorial and frequently carried *EGFR* amplification, but they did not perform WES to determine the status of the mutations mentioned above. Furthermore, they showed that *PDGFRA* amplifications are a hallmark of pedHGG_RTK1 tumors, which often arise subsequently to cranial irradiation or in the context of a replication repair deficiency such as Lynch syndrome.^39,40^ Consistent with the absence of pedHGG_RTK1 gliomas, *PDGFRA* mutations/amplifications, which are found in up to 16% of hemispheric pedHGG,^26,41^ were scarce in our cohort. Our pediatric GC collection was characterized by structural aberrations in chromosome 6 across various WHO 2021 CNS tumor (sub-)types as well. Chromosome 6 alterations have not yet been described in pedHGG in general, and their significance needs to be investigated in further studies on the various subtypes. Taken together, the dominance of the methylome-subclasses pedHGG_RTK2A/B and pedHGG_A/B, and the absence of certain other subclasses, which we could reproduce in an independent, reclassified case series by Broniscer et al.,^12^ as well as the exceptional genetic repertoire of pediatric-type diffuse gliomas with GC phenotype underlines a distinct molecular profile in these tumors. This is in contrast to earlier reports primarily due to smaller case numbers, an earlier version of the methylation classifier, and a lack of comprehensive exome sequencing.

Specific treatment recommendations for GC in children are lacking. Therapy proposals within case reports or small case series were based on approaches in unselected diffuse gliomas, but their impact in GC remained unclear.^42,43^ In HGC, we observed a marked difference in PFS between upfront therapy modalities favoring a combination of chemo- and radiotherapy versus chemotherapy alone. However, the OS of the whole cohort and of different subgroups was not significantly influenced by the initial treatment modalities, possibly due to the fact that irradiation, which had been omitted primarily, in most cases was subsequently administered after disease progression. Overall, our data showed that children with GC were treated very heterogeneously. Since these tumors behave very aggressively and differ clinically as well as molecularly from unselected hemispheric pedHGG, GC might warrant specific treatment approaches but due to its rarity prospective trials are difficult to conduct. We advocate that GC should be given special consideration in future studies on pedHGG, for example, by introducing an additional radiological label for GC-like diffuse glioma in clinical trials or by focusing on the radiological growth pattern in studies on methylation-based subclasses. By implementing a GC label, more attention would be paid to the clinical impact of this extensively infiltrating growth pattern, and further insights can be gained about this phenotype. In general, therapeutic approaches in GC should consider individual genetic alterations and should evaluate systematically the efficacy of tailored treatments (eg, EGFR inhibitors) as part of the multimodal approach. In our collective, the significance of such a targeted therapy could not be assessed due to the small number of cases. Clinical trial participation based on a GC phenotype, presence of specific genetic alterations, or on methylome would make study results more comparable and provide person-centered treatment options for GC patients, who have often been excluded from participation in clinical trials in the past.^44^

In conclusion, we provide evidence for molecular signatures enriched in pediatric-type diffuse gliomas with GC phenotype: predominance of pedHGG_RTK2A/B and pedHGG_A/B methylation-defined subclasses as well as of *EGFR*, *BCOR*, and chromosome 6 alterations; the absence of the pedHGG_RTK1A/B/C subclasses and of *CDKN2A/B* and *PDGFRA* alterations. Furthermore, we assembled comprehensive clinical and radiological characterization and could identify certain prognostic parameters (eg, histopathological grading in the whole cohort, contrast enhancement in HGC, the presence of *TP53* mutations in the pedHGG_H3-/IDH-wt subgroup). Taken together, these findings expand the current knowledge of GC and provide insight into disease biology of extensively infiltrating gliomas in children.

## Supplementary material

Supplementary material is available online at *Neuro-Oncology* (https://academic.oup.com/neuro-oncology).

noae080_suppl_Supplementary_Data

## Data Availability

Original data originated in the course of this study, that are not accessible by the publication, will be made available personally upon reasonable request.

## Prior presentation

• Preliminary results of this study have in part been presented as a poster at the 20th International Symposium on Pediatric Neuro-Oncology, June 12th –15th, 2022, Hamburg, Germany.

• Results have been presented as a poster in part at the 2023 Society for Neuro-Oncology Neuro-Oncology Pediatric Research Conference, June 22nd –24th, 2023, Washington, DC, USA.
