# Supplementary material for: Gliomatosis cerebri in children: A poor prognostic phenotype of diffuse gliomas with a distinct molecular profile
Source: Neuro Oncol. 2024 May 8;26(9):1723–37. doi: 10.1093/neuonc/noae080 (PMC11376460; doi:10.1093/neuonc/noae080)
Supplement: noae080_suppl_Supplementary_Data [file noae080_suppl_supplementary_data.zip › Suppl figures and Tables/Supplementary_Table_and_Figure_Legends_N-O-D-23-00918R1.docx]

**Supplementary Figure 1: GC vs. partially resected hemispheric pedHGG**

(A) Comparison of overall survival (OS) between the pediatric GC cases with high-grade features (HGC) and a cohort (n=108) of biopsied/partially resected hemispheric high-grade gliomas in children (pedHGG) extracted from the prospective German HIT HGG therapy trials. Age limits were adjusted to those of the GC cohort. There was a significantly worse OS in the HGC subgroup. In the background, the overall survival of (sub-)totally resected hemispheric pedHGG (n=202) is shown gray dotted. (B) Multivariate analysis of the HGC subgroup (n=89) and the control cohort of pedHGG including total/subtotal resected and partially resected/biopsied tumors (in total n=310). Significant values are in bold.

**Supplementary Figure 2: DNA copy number of pediatric GC**

Heatmap representation of segmented DNA copy number for 49 pediatric GC samples derived from methylation array data (dark red, amplification; red, gain; dark blue, deletion; blue, loss). Samples are arranged in columns clustered by gene-level data across the whole genome. Clinicopathological and molecular annotations are provided as bars according to the included key.

**Supplementary Figure 3: Re-classification of published GC datasets**

(A) t-statistic based stochastic neighbor embedding (t-SNE) projection of a combined methylation dataset comprising the pediatric GC cases from Broniscer et al. (circled, n=14)^12^ plus a reference set of glioma subtypes (n=2305). The first two projections are plotted on the x and y axes, with samples represented by dots colored by subtype as labelled on the figure. (B) t-SNE projection of a combined methylation dataset comprising the adult GC cases from Herrlinger et al. (circled, n=20)^11^ plus a reference set of glioma subtypes (n=2305). The first two projections are plotted on the x and y axes, with samples represented by dots colored by subtype as labelled in the figure. (C–D) Representative DNA copy number plot for the cases STJ_024 and DKFZ_A02, respectively, derived from methylation array data, with log2 ratios plotted (y axis) against genomic location by chromosome (x axis), and colored red for gain, and blue for loss. Above – whole genome; below – chromosome 6.

**Supplementary Figure 4: DNA copy number of published GC datasets**

Heatmaps representation of segmented DNA copy number for 18 pediatric GC cases (A) from Broniscer et al.^12^ number for 25 adult GC cases (B) from Herrlinger et al.^11^. Both datasets are derived from DNA methylation array data (dark red, amplification; red, gain; dark blue, deletion; blue, loss). Samples are arranged in columns clustered by gene-level data across the whole genome. Clinicopathological and molecular annotations are provided as bars according to the included key.

**Supplementary Figure 5: Comparison of the three main subclasses**

Comparison of clinical and molecular parameter of the three main subclasses of diffuse pediatric-type high-grade glioma, H3-wildtype and IDH-wildtype including pedHGG_A/B, a novel molecular subclass according to MNP12.5. In the first row, representative axial MRI images of each subclass are shown.

**Supplementary Table 1: Radiological characteristics**

Abbreviations: magnetic resonance imaging (MRI); ^†^hydrocephalus at diagnosis was mild in most of these cases.

**Supplementary Table 2****A: Clinical characteristics of the whole cohort**

Abbreviations: acute lymphoblastic leukemia (ALL), cerebrospinal fluid (CSF), Gray (Gy), magnetic resonance imaging (MRI), primitive neuro-ectodermal tumor (PNET), temozolomide (TMZ); ^1^confirmation through autopsy since the patient succumbed rapidly after initial radiological evaluation; ^2^histopathology findings compatible with diffuse glioma without specification of histopathological grade; ^3^only TMZ; ^4^TMZ in combination with other cytotoxic agents; ^5^chemotherapeutic regimes without TMZ; in ‘TMZ-Multi’ and ‘Other’ targeted therapies were administered in individual patients; ^6^combination of irradiation and chemotherapy encompassing synchronic or sequential administration; ^7^including Cilengitide (n=3), Pembrolizumab (n=3), Imatinib (n=2), mTOR-inhibition (n=3), Sorafenib (n=1), Pomalidomide (n=1), MEMMAT regime (n=1); *all four previous malignancies were treated with radiotherapy; ^†^multiple entries possible.

**Supplementary Table 2****B:** **Clinical course and outcome of the whole cohort**

^‡^In three cases progression status was not available.

**Supplementary Table 3: Multivariate Cox Regression analysis for progression free- and overall survival of the whole cohort**

*p*-values under the threshold for statistical significance (<0.05) are in bold; abbreviations: hazard ratio (HR), confidence interval (CI); ^1^*p*-value; *reference parameter.

**Supplementary Table 4:** **Multivariate Cox Regression analysis for progression free and overall survival of the subgroup of diffuse pediatric-type high-grade glioma, H3-wildtype and IDH-wildtype**

*p*-values under the threshold for statistical significance (<0.05) are in bold; abbreviations: hazard ratio (HR), confidence interval (CI); ^1^*p*-value; *reference parameter.
