# Supplementary material for: Gliomatosis cerebri in children: A poor prognostic phenotype of diffuse gliomas with a distinct molecular profile
Source: Neuro Oncol. 2024 May 8;26(9):1723–37. doi: 10.1093/neuonc/noae080 (PMC11376460; doi:10.1093/neuonc/noae080)
Supplement: noae080_suppl_Supplementary_Data [file noae080_suppl_supplementary_data.zip › Suppl figures and Tables/Suppl_Table1.docx]

| **Number of affected cerebral lobes** | | | **n=104** | **100%** |
| --- | --- | --- | --- | --- |
|  | 3 lobes |  | 44 | 42.4% |
|  | 4 lobes |  | 30 | 28.9% |
|  | 5 lobes |  | 21 | 20.2% |
|  | 6 lobes |  | 7 | 6.7% |
|  | 7 lobes |  | 1 | 0.9% |
|  | 8 lobes |  | 1 | 0.9% |
| **Individual affected lobes** | | | **n=104** | **100%** |
|  | Frontal lobe | uni-/bilateral | 65 / 39 | 62.5% / 37.5% |
|  | Temporal lobe | uni-/bilateral | 69 / 33 | 66.4% / 31.7% |
|  | Parietal lobe | uni-/bilateral | 67 / 15 | 64.4% / 14.4% |
|  | Occipital lobe | uni-/bilateral | 40 / 1 | 38.5% / 0.9% |
|  | Unilateral cerebral involvement | | 48 | 46.2% |
|  | Bihemispheric cerebral involvement | | 56 | 53.8% |
| **Structures additionally involved** | | | **n=104** | **100%** |
|  | Thalamus | uni-/bilateral | 48 / 30 | 46.1% / 28.9% |
|  | Basal ganglia | uni-/bilateral | 57 / 8 | 54.8% / 7.7% |
|  | Infratentorial involvement |  | 35 | 33.7% |
| **Additional radiological information** | | | **n=104** | **100%** |
|  | Contrast enhancement | ≤25% of tumor mass | 42 | 40.4% |
|  |  | 26-50% of tumor mass | 4 | 3.8% |
|  |  | 51-75% of tumor mass | 3 | 2.9% |
|  |  | >75% of tumor mass | 0 | 0.0% |
|  |  | No contrast enhancement | 50 | 48.1% |
|  |  | No contrast agent applied | 5 | 4.8% |
|  | Necrosis | ≤25% of tumor mass | 12 | 11.6% |
|  |  | >26% of tumor mass | 1 | 0.9% |
|  |  | No necrosis | 90 | 86.6% |
|  |  | Not specified | 1 | 0.9% |
|  | Hydrocephalus^†^ |  | 11 | 10.6% |

**Supplementary Table 1**
