# Supplementary material for: Gliomatosis cerebri in children: A poor prognostic phenotype of diffuse gliomas with a distinct molecular profile
Source: Neuro Oncol. 2024 May 8;26(9):1723–37. doi: 10.1093/neuonc/noae080 (PMC11376460; doi:10.1093/neuonc/noae080)
Supplement: noae080_suppl_Supplementary_Data [file noae080_suppl_supplementary_data.zip › Suppl figures and Tables/Suppl_Table2A_clear.docx]

| **Baseline characteristics** | | | | **n=104** | **100%** |
| --- | --- | --- | --- | --- | --- |
|  | Sex | Male | | 69 | 66.3% |
|  |  | Female | | 35 | 33.7% |
|  | Age at diagnosis | Median | | 11.8 years |  |
|  |  | Range | | 1.3-18.8 years |  |
|  | Predisposing/preceding diseases | | |  |  |
|  |  | ALL* | | 2 | 1.9% |
|  |  | Medulloblastoma* | | 1 | 0.9% |
|  |  | PNET* | | 1 | 0.9% |
|  |  | *TP53* germline mutation | | 1 | 0.9% |
|  |  | *MSH2* germline mutation | | 1 | 0.9% |
| **Initial symptoms** | |  | | **n=205^†^** | **100%** |
|  | Seizure |  | | 48 | 23.4% |
|  | Headache |  | | 37 | 18.1% |
|  | Sensorimotor deficits |  | | 35 | 17.1% |
|  | Visual symptoms |  | | 26 | 12.7% |
|  | Nausea/vomiting |  | | 23 | 11.2% |
|  | Others |  | | 36 | 17.5% |
| **Type of initial surgery** | | | | **n=104** | **100%** |
|  | Biopsy |  | | 79 | 76.0% |
|  | Partial resection |  | | 24 | 23.1% |
|  | No surgery^1^ |  | | 1 | 0.9% |
| **Neuropathological assessment** | | | | **n=104** | **100%** |
|  | Central histopathological review | | | 81 | 77.9% |
|  | WHO grade | II | | 12 | 11.5% |
|  |  | III | | 68 | 65.4% |
|  |  | IV | | 21 | 20.2% |
|  |  | Not specified^2^ | | 3 | 2.9% |
| **Initial therapy** | | | | **n=104** | **100%** |
|  | Radiotherapy | | | 79 | 75.9% |
|  | Irradiation modality | Focal | | 42 | 40.4% |
|  |  | Whole brain | | 12 | 11.5% |
|  |  | Whole brain + boost | | 15 | 14.4% |
|  |  | Craniospinal | | 3 | 2.9% |
|  |  | Unknown irradiation modality | | 7 | 6.7% |
|  | Total irradiation dose | ≤50 Gy | | 16 | 15.4% |
|  |  | >50 Gy | | 61 | 58.6% |
|  |  | Unknown radiation dose | | 2 | 1.9% |
|  | Chemotherapy | | | 94 | 90.4% |
|  |  | TMZ-Mono^3^ | | 52 | 50.0% |
|  |  | TMZ-Multi^4^ | | 14 | 13.5% |
|  |  | Other^5^ | | 28 | 26.9% |
|  | Combined modality treatment^6^ | | | 73 | 70.2% |
|  | Targeted therapy (first line / after progression) | | |  |  |
|  |  | | EGFR inhibitor | 7 / 7 | 6.7% / 6.7 % |
|  |  | | VEGF inhibitor | 5 / 14 | 4.8 / 13.5 % |
|  |  | | Other^7^ | - / 11 | 1.9% / 10.6% |
|  | No upfront therapy |  | | 4 | 3.8% |

**Supplementary Table 2A**
