# Supplementary material for: Gliomatosis cerebri in children: A poor prognostic phenotype of diffuse gliomas with a distinct molecular profile
Source: Neuro Oncol. 2024 May 8;26(9):1723–37. doi: 10.1093/neuonc/noae080 (PMC11376460; doi:10.1093/neuonc/noae080)
Supplement: noae080_suppl_Supplementary_Data [file noae080_suppl_supplementary_data.zip › Suppl figures and Tables/Suppl_Table2B.docx]

| **Follow-up** | | | **n=104** |  |
| --- | --- | --- | --- | --- |
|  | (in months) | Median | 15.5 |  |
|  |  | Range | 2.3-138.8 |  |
| **Progression status** | | | **n=104** | **100 %** |
|  | No progression |  | 4 | 3.8% |
|  | Progression |  | 97 | 93.3% |
|  | Unknown event status |  | 3 | 2.9% |
| **Vital status** | | | **n=104** | **100%** |
|  | Alive |  | 7 | 6.8% |
|  |  | >5-year survival | 4 | 3.8% |
|  | Dead due to disease progression | | 93 | 89.4% |
|  | Lost to follow-up |  | 4 | 3.8% |
| **Prediagnostic symptomatic interval** | | | **n=104** |  |
|  | (in weeks) | Median | 8 |  |
|  | | Interquartile range | 3-15 |  |
| **Progression-free survival** | | | **n=101^‡^** |  |
|  | (in months) | Median | 8.6 |  |
|  | | Interquartile range | 4.3-14.0 |  |
| **Overall survival** | | | **n=104** |  |
|  | (in months) | Median | 15.5 |  |
|  | | Interquartile range | 10.9-27.7 |  |
|  |  | 1-year survival |  | 68% |
|  | | 2-year survival |  | 28% |

**Supplementary Table 2B**
