# Supplementary material for: Gliomatosis cerebri in children: A poor prognostic phenotype of diffuse gliomas with a distinct molecular profile
Source: Neuro Oncol. 2024 May 8;26(9):1723–37. doi: 10.1093/neuonc/noae080 (PMC11376460; doi:10.1093/neuonc/noae080)
Supplement: noae080_suppl_Supplementary_Data [file noae080_suppl_supplementary_data.zip › Suppl figures and Tables/Suppl_Table3_clear.docx]

|  | | |  | **Progression-free survival** | |  | **Overall survival** |  |
| --- | --- | --- | --- | --- | --- | --- | --- | --- |
| **Variables and categories** | | | **n=89** | **HR (95% CI)** | ***p*^1^** | **n=92** | **HR (95% CI)** | ***p*^1^** |
| Number of affected cerebral lobes | | |  |  |  |  |  |  |
|  | <5* vs. ≥5 | | 65 vs. 24 | 0.71 (0.37-1.34) | 0.29 | 66 vs. 26 | 0.81 (0.44-1.52) | 0.51 |
| Bihemispheric involvement | | |  |  |  |  |  |  |
|  | Unilateral* vs. bihemispheric | | 42 vs. 47 | 0.94 (0.51-1.71) | 0.83 | 43 vs. 49 | 0.82 (0.45-1.51) | 0.53 |
| Thalamic involvement | | |  |  |  |  |  |  |
|  | No/unilateral* vs. bilateral | | 64 vs. 25 | 1.72 (0.92-3.24) | 0.09 | 66 vs. 26 | 1.63 (0.85-3.14) | 0.14 |
| Involvement of basal ganglia | | |  |  |  |  |  |  |
|  | No/unilateral* vs. bilateral | | 85 vs. 4 | 0.24 (0.06-1.02) | 0.06 | 87 vs. 5 | 1.12 (0.35-3.58) | 0.85 |
| Infratentorial involvement | | |  |  |  |  |  |  |
|  | No involvement* vs. involvement | | 59 vs. 30 | 1.48 (0.8-2.75) | 0.21 | 60 vs. 32 | 1.15 (0.63-2.11) | 0.65 |
| Contrast enhancement | | |  |  |  |  |  |  |
|  | No enhancement* vs. enhancement | | 46 vs. 43 | 1.79 (1.03-3.11) | **0.04** | 46 vs. 46 | 0.87 (0.49-1.57) | 0.65 |
| Gender | | |  |  |  |  |  |  |
|  | Male* vs. female | | 58 vs. 31 | 0.8 (0.47-1.36) | 0.41 | 60 vs. 32 | 0.62 (0.35-1.08) | 0.09 |
| Age (in years) | | |  |  |  |  |  |  |
|  | <4 |  | 6 | 0.57 (0.21-1.58) | 0.28 | 6 | 0.47 (0.15-1.48) | 0.19 |
|  | 4-10 |  | 25 | Ref. |  | 25 | Ref. |  |
|  | >10 |  | 58 | 1.15 (0.65-2.04) | 0.64 | 61 | 0.90 (0.51-1.57) | 0.70 |
| WHO grade | | |  |  |  |  |  |  |
|  | II | | 9 | 0.4 (0.16-0.96) | **0.04** | 9 | 0.44 (0.18-1.11) | 0.08 |
|  | III | | 62 | Ref. |  | 63 | Ref. |  |
|  | IV | | 18 | 1.94 (0.94-4.02) | 0.07 | 20 | 3.41 (1.61-7.21) | **0.001** |
| Upfront therapy | | |  |  |  |  |  |  |
|  | Only radiotherapy | | 4 | 0.18 (0.05-0.65) | **0.008** | 4 | 0.51 (0.11-2.44) | 0.38 |
|  | Only chemotherapy | | 16 | Ref. |  | 17 | Ref. |  |
|  | Chemo- and radiotherapy | | 69 | 0.14 (0.07-0.3) | **<0.001** | 71 | 1.15 (0.59-2.27) | 0.66 |
| Re-Irradiation | | |  |  |  |  |  |  |
|  | Yes vs. no | | NA | NA | NA | 8 vs. 96 | 0.94 (0.37-2.35) | 0.89 |

**Supplementary Table 3**
