# Supplementary material for: Gliomatosis cerebri in children: A poor prognostic phenotype of diffuse gliomas with a distinct molecular profile
Source: Neuro Oncol. 2024 May 8;26(9):1723–37. doi: 10.1093/neuonc/noae080 (PMC11376460; doi:10.1093/neuonc/noae080)
Supplement: noae080_suppl_Supplementary_Data [file noae080_suppl_supplementary_data.zip › Suppl figures and Tables/Suppl_Table4.docx]

|  | | |  | **Progression-free survival** | |  | **Overall survival** |  |
| --- | --- | --- | --- | --- | --- | --- | --- | --- |
| **Variables and categories** | | | **n=27** | **HR (95% CI)** | ***p*^1^** | **n=27** | **HR (95% CI)** | ***p*^1^** |
| Age (in years) | | |  |  |  |  |  |  |
|  | <10* vs. ≥10 |  | 9 vs. 18 | 0.77 (0.19-3.07) | 0.71 | 9 vs. 18 | 0.27 (0.06-1.20) | 0.09 |
| Methylation subclass | | |  |  |  |  |  |  |
|  | pedHGG_MYCN | | 5 | 0.87 (0.19-4.02) | 0.85 | 5 | 0.43 (0.08-2.16) | 0.3 |
|  | pedHGG_A/B | | 5 | 1.15 (0.41-3.28) | 0.79 | 5 | 0.84 (0.26-2.65) | 0.76 |
|  | pedHGG_RTK2A/B | | 17 | Ref. |  | 17 | Ref. |  |
| *TP53*-status | | |  |  |  |  |  |  |
|  | No alteration* vs. alteration | | 19 vs. 8 | 3.98 (1.26-12.5) | **0.018** | 19 vs. 8 | 6.15 (1.59-23.7) | **0.008** |

**Supplementary Table 4**
