# Supplementary figures and images for: Gliomatosis cerebri in children: A poor prognostic phenotype of diffuse gliomas with a distinct molecular profile
Source: Neuro Oncol. 2024 May 8;26(9):1723–37. doi: 10.1093/neuonc/noae080 (PMC11376460; doi:10.1093/neuonc/noae080)

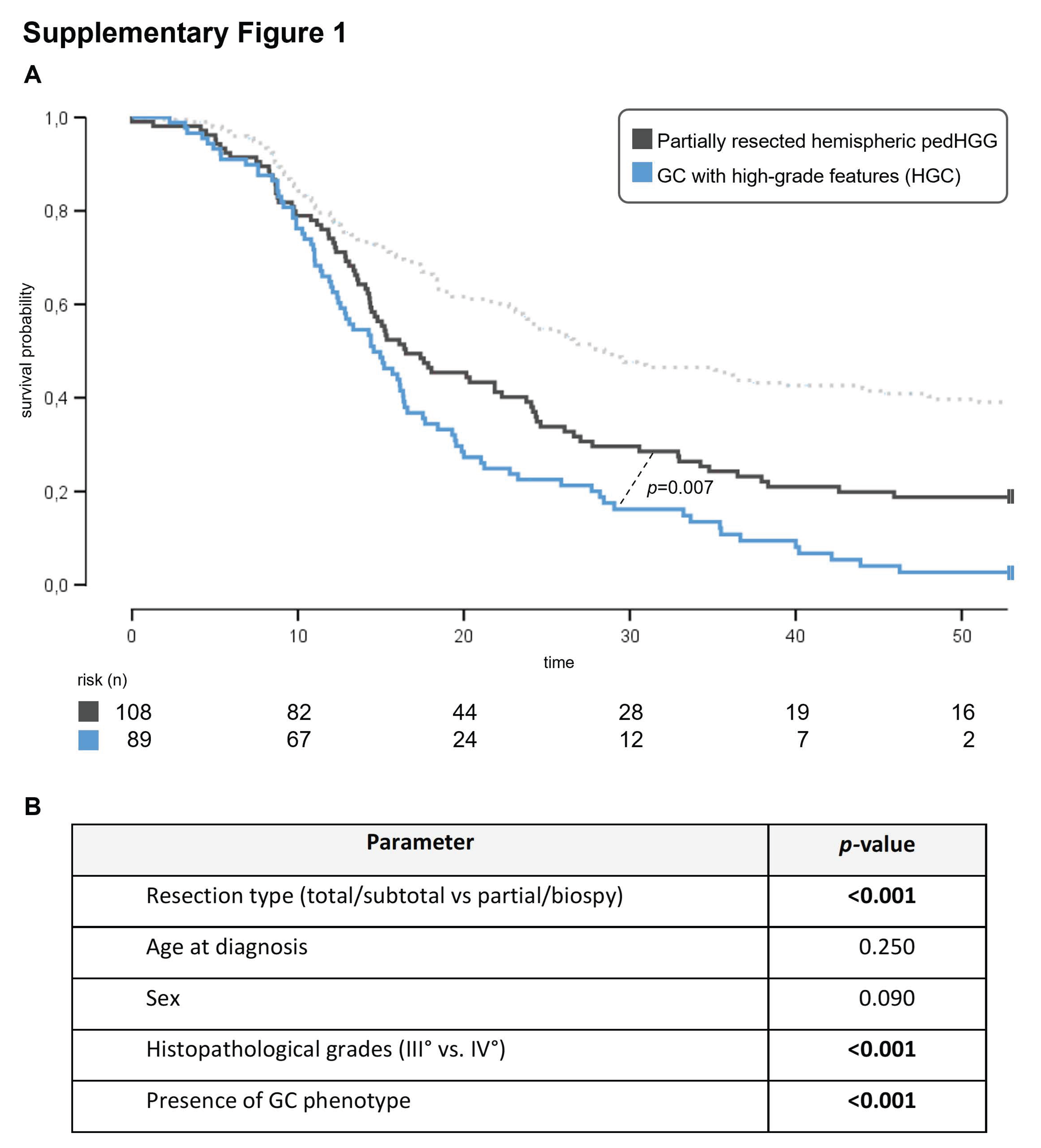

Supplement: noae080_suppl_Supplementary_Data [file noae080_suppl_supplementary_data.zip › Suppl figures and Tables/Suppl.Fig_1.tiff]

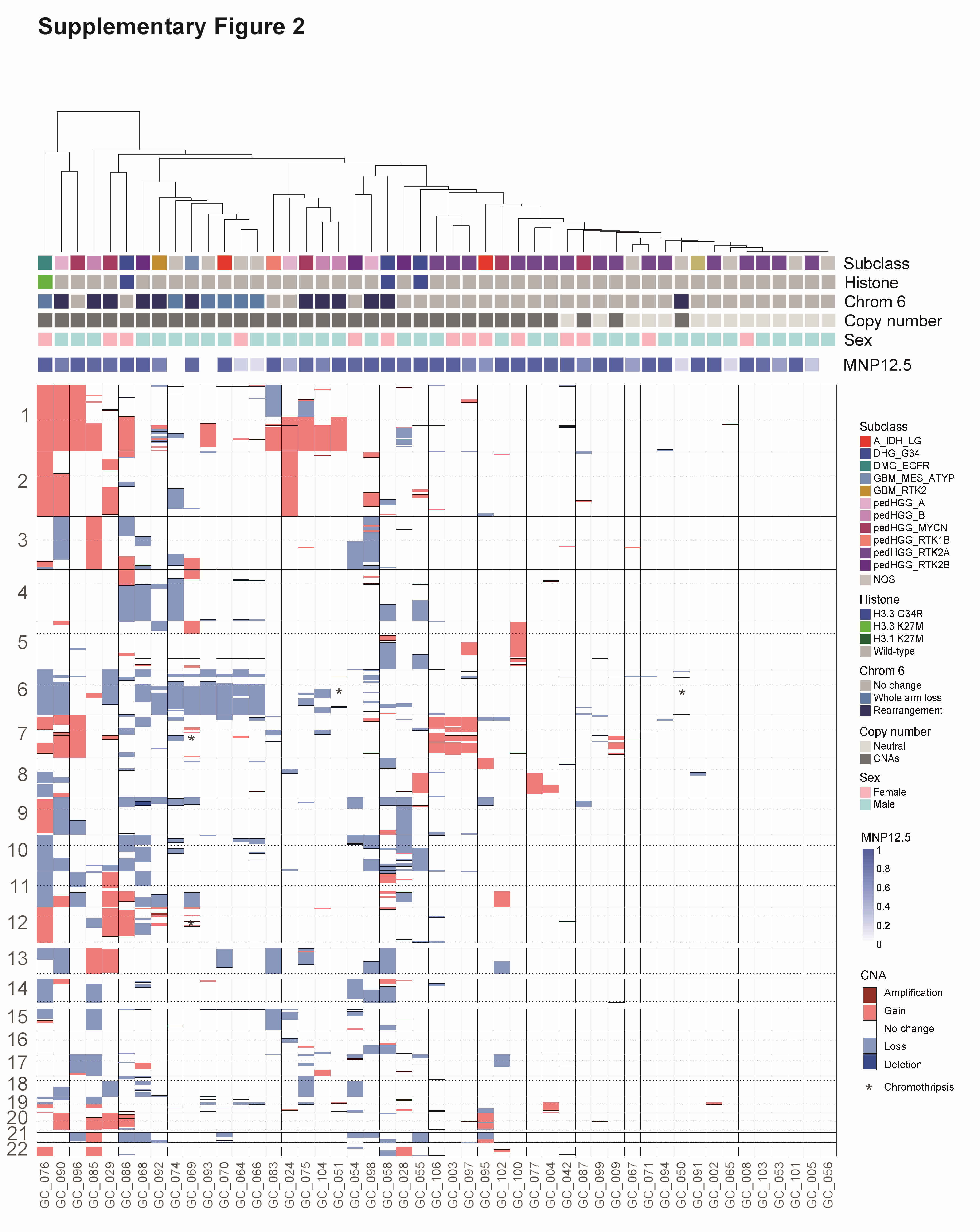

Supplement: noae080_suppl_Supplementary_Data [file noae080_suppl_supplementary_data.zip › Suppl figures and Tables/Suppl.Fig_2.tiff]

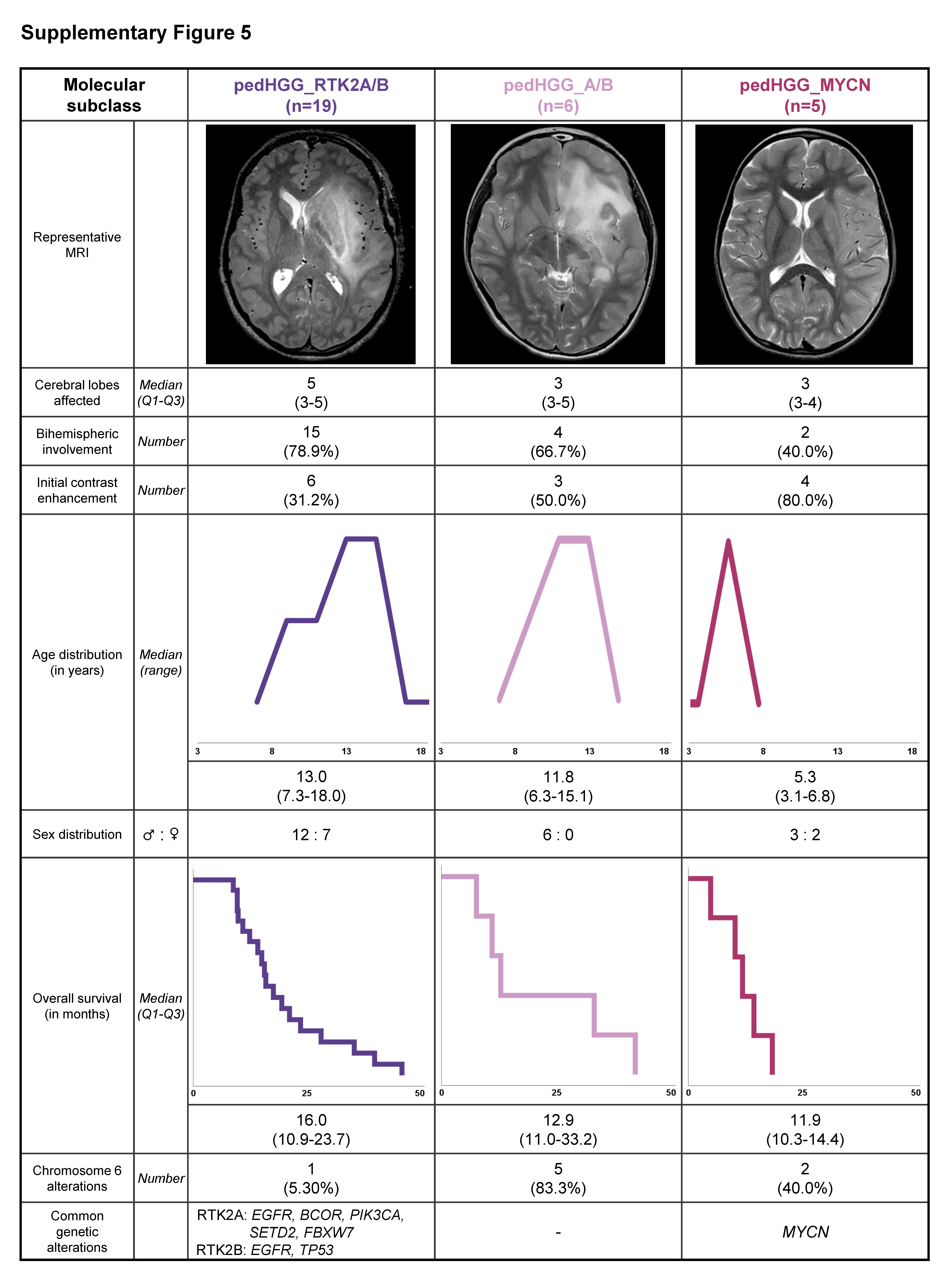

Supplement: noae080_suppl_Supplementary_Data [file noae080_suppl_supplementary_data.zip › Suppl figures and Tables/Suppl.Fig_5.tiff]

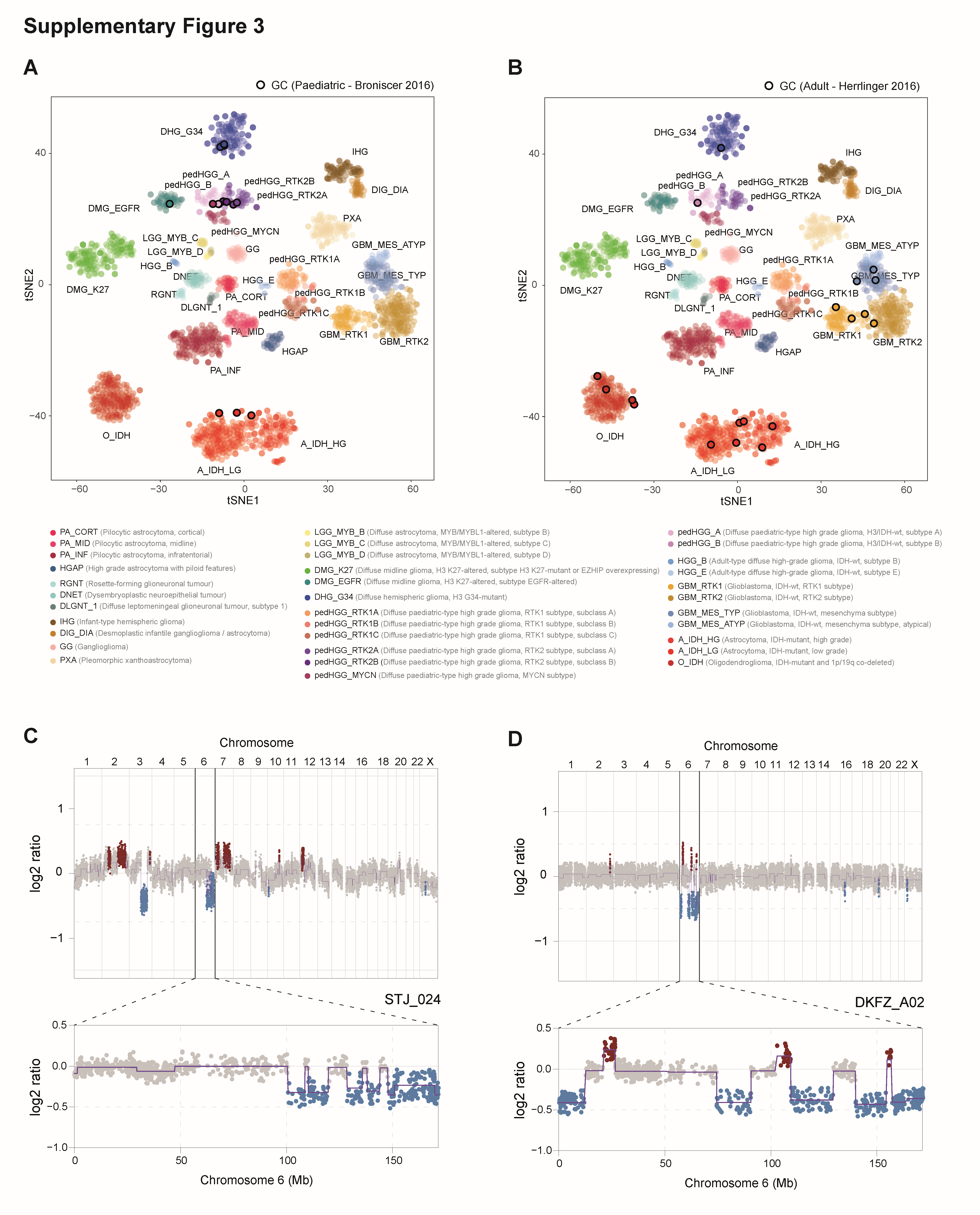

Supplement: noae080_suppl_Supplementary_Data [file noae080_suppl_supplementary_data.zip › Suppl figures and Tables/Suppl_Fig_3.tiff]

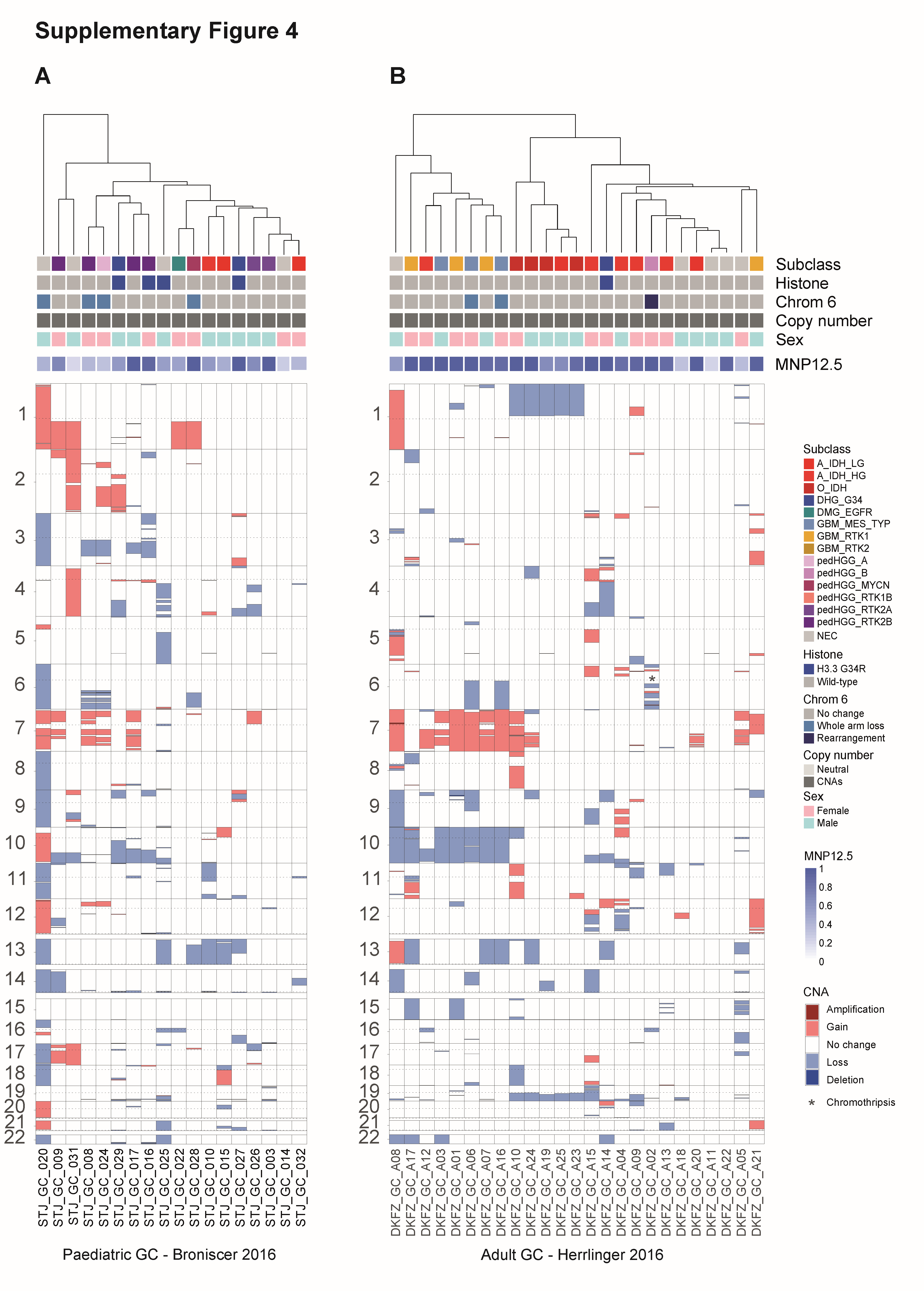

Supplement: noae080_suppl_Supplementary_Data [file noae080_suppl_supplementary_data.zip › Suppl figures and Tables/Suppl_Fig_4.tiff]
